# Meditative Movement as a Treatment for Pulmonary Dysfunction in Flight Attendants Exposed to Second-Hand Cigarette Smoke: Study Protocol for a Randomized Trial

**DOI:** 10.3389/fpsyt.2016.00038

**Published:** 2016-03-22

**Authors:** Peter Payne, David Zava, Steven Fiering, Mardi Crane-Godreau

**Affiliations:** ^1^Microbiology and Immunology, Geisel School of Medicine at Dartmouth, Hanover, NH, USA; ^2^ZRT Laboratory, Beaverton, OR, USA

**Keywords:** meditative movement, Qigong, flight attendants, second-hand cigarette smoke, pulmonary dysfunction, COPD, autonomic nervous system, inflammation

## Abstract

A study protocol is presented for the investigation of meditative movement (MM) as a treatment for pulmonary dysfunction in flight attendants (FA) who were exposed to second-hand cigarette smoke while flying before the smoking ban. The study will have three parts, some of which will run concurrently. The first is a data gathering and screening phase, which will gather data on pulmonary and other aspects of the health of FA, and will also serve to screen participants for the other phases. Second is an exercise selection phase, in which a variety of MM exercises will be taught, over a 16-week period, to a cohort of 20 FA. A subset of these exercises will be selected on the basis of participant feedback on effectiveness and compliance. Third is a 52-week randomized controlled trial to evaluate the effectiveness of a digitally delivered form of the previously selected exercises on a group of 20 FA, as compared with an attention control group. Outcome measures to be used in all three parts of the study include the 6-min walk test as a primary measure, as well as a range of biomarkers, tests, and questionnaires documenting hormonal, cardio-respiratory, autonomic, and affective state. This study is registered at ClinicalTrials.gov. Identifier: NCT02612389.

## Background

### COPD and Its Co-Morbidities in FA

**Figure 1:**
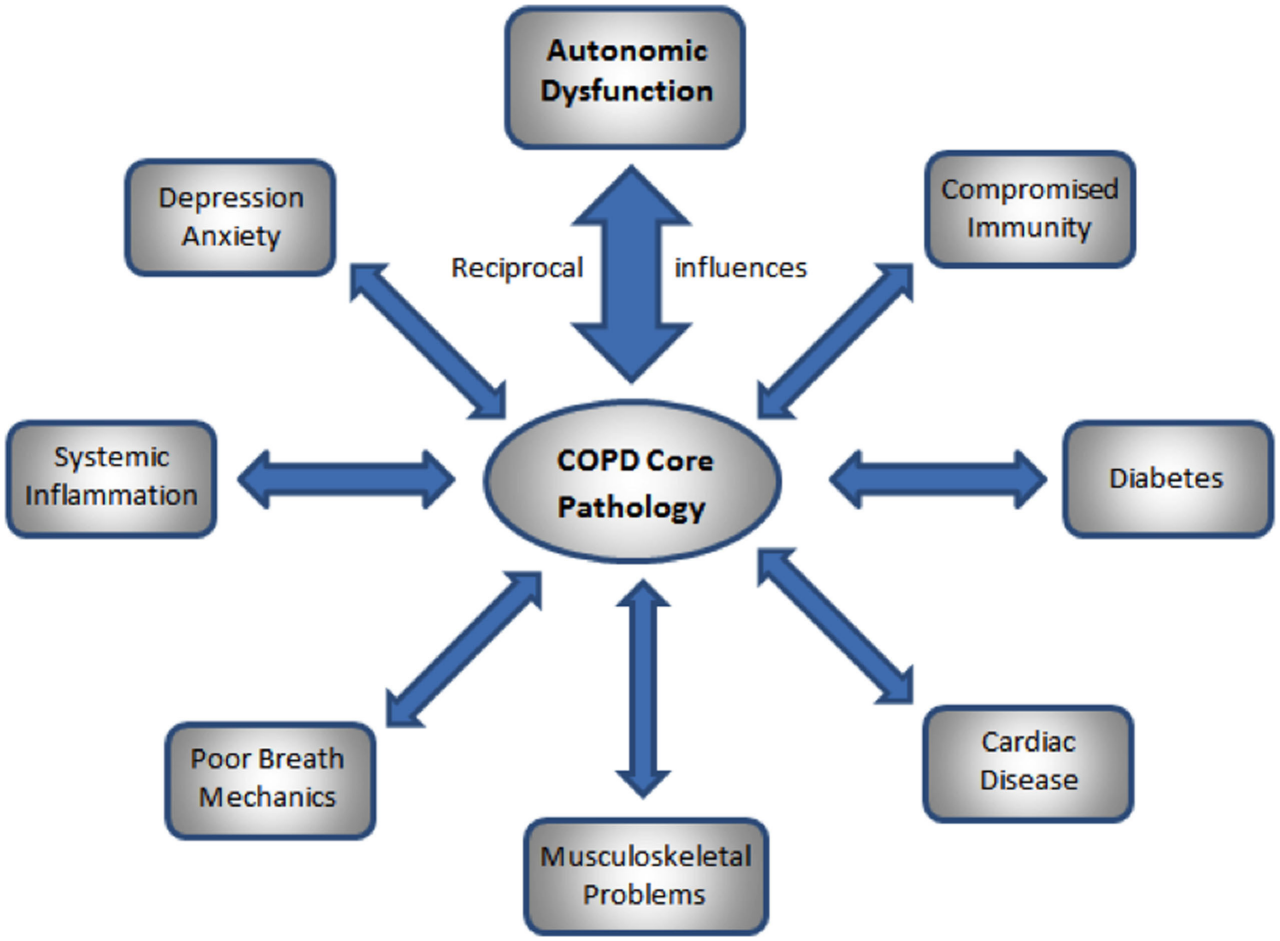
**Reciprocal influences of core COPD pathology and the co-morbidities of COPD**.

Chronic obstructive pulmonary disease (COPD) is a major cause of morbidity and mortality worldwide; the primary cause of COPD is exposure to cigarette smoke. Those exposed to second-hand cigarette smoke (SHCS) are at increased risk for respiratory, cardiovascular, and other organ system disease that are similar to those suffered by smokers (1). Many flight attendants (FA) who flew before the ban on smoking in commercial aircraft, and who were thus exposed to SHS, have abnormal pulmonary function consistent with mild COPD, despite not meeting the GOLD criteria of reduced FEV1/FVC ratio (2). They also show increased rates of many of the co-morbidities associated with COPD, such as chronic bronchitis, cardiovascular disease, skin cancer, and depression and anxiety (3). The co-morbidities of COPD exacerbate and are exacerbated by the COPD (4). Thus, consideration of the co-morbidities of COPD is an essential aspect of its treatment (4). Significant co-morbidities include cardiovascular disease (4); depression and anxiety (5); muscle weakness, osteoporosis, skin and lung cancer, and diabetes (4); as well as autonomic disturbance (6), systemic inflammation and cachexia (4); frequent hypoxia, dyspnea, and disturbed breathing patterns (7); and disturbed posture and movement (8); see Figure 1.

### The Autonomic Nervous System in COPD

**Figure 2:**
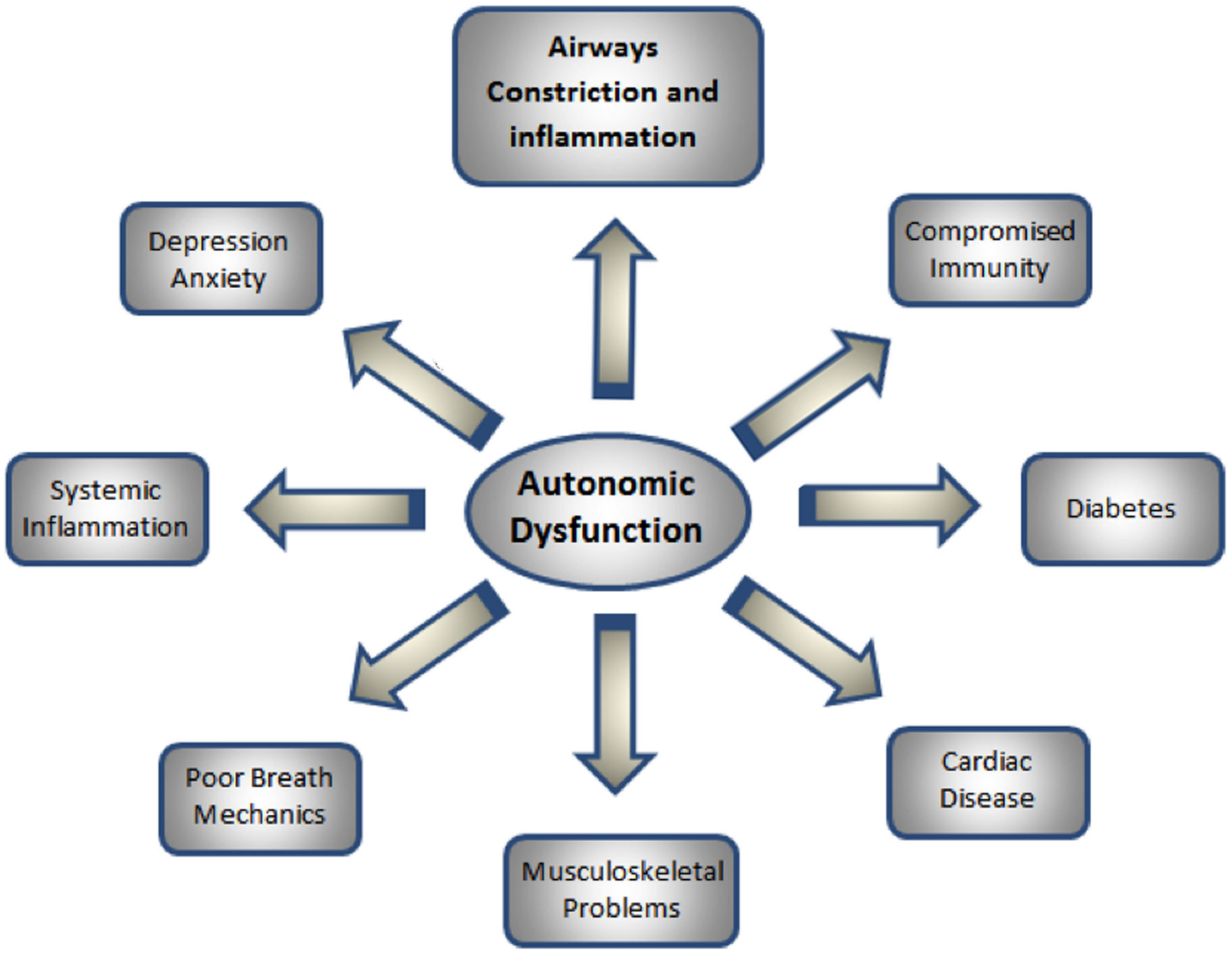
**Relationships of autonomic dysfunction to the co-morbidities of COPD**.

Autonomic dysfunction (AD) is known to be associated with COPD and most of its co-morbidities (6, 9–15); see Figure 2. Most of the diseases known to be co-morbidities of COPD may be caused or exacerbated by AD; for instance, cardiovascular disease (15, 16); hypoxia (17, 18); disturbed respiratory patterns (7, 19, 20); disturbed posture and movement patterns (21, 22); diabetes (17); immune function (4); airways restriction (6, 23); and anxiety and depression (12).

### Meditative Movement

Qigong, a traditional Chinese health practice, has been used in China for hundreds of years in treating those with respiratory disease (24). Recently, a case has been made for a novel category of exercise “meditative movement,” (MM) of which Qigong, Tai Chi, and Hatha Yoga are examples (25). Significantly, MM is hypothesized to act via its effect on the autonomic nervous system (ANS) (26, 27), as well as on neuromuscular control (28, 29), musculoskeletal condition (30), and mental state (31).

Meditative movement has been shown to be an effective intervention for COPD (30, 32–36) and equivalent or superior to conventional pulmonary rehabilitation in benefiting several symptoms of COPD (37).

**Figure 3:**
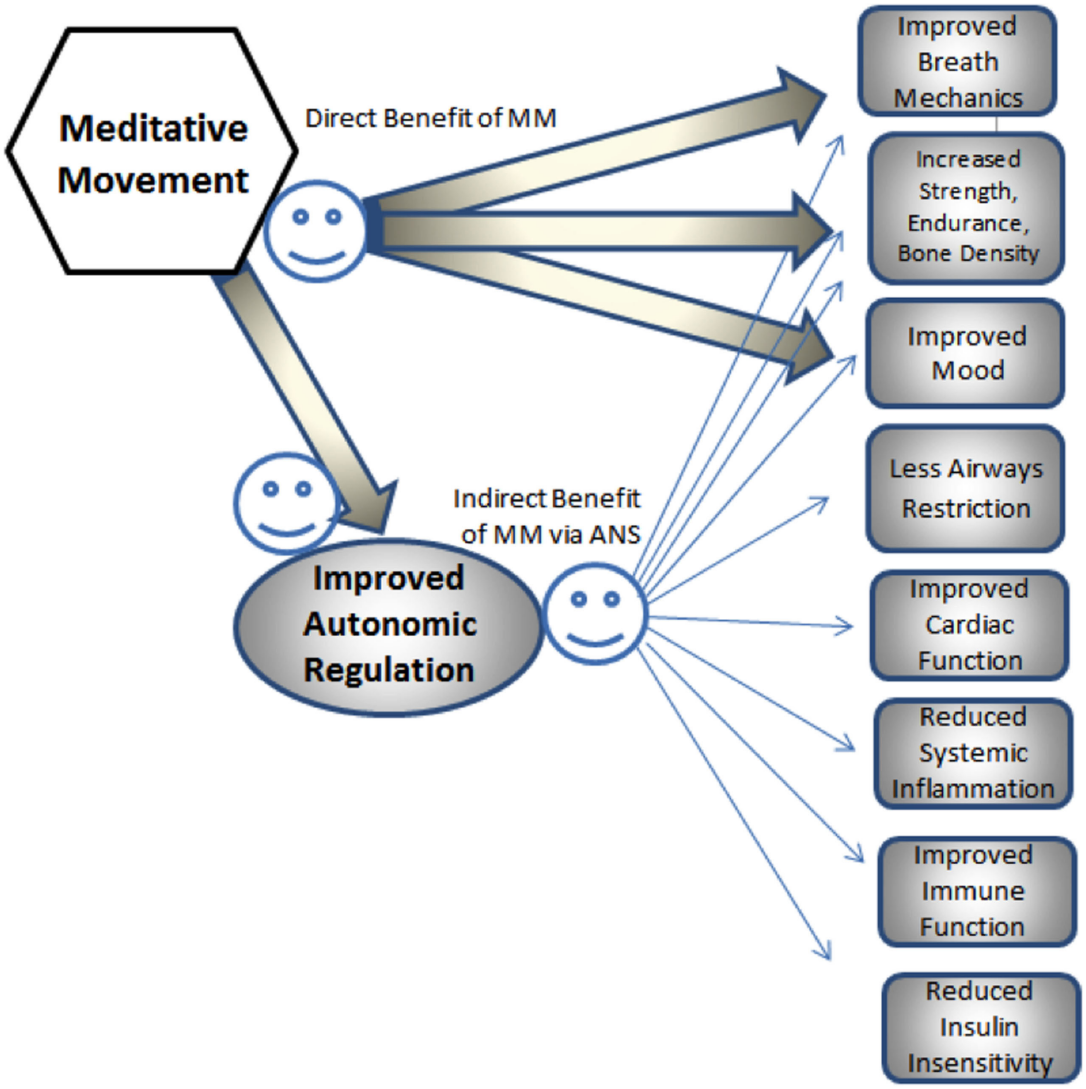
**Hypothesized pathways of direct and indirect influence of MM on co-morbidities of COPD**.

Meditative movement may also be of benefit in many co-morbidities of COPD, such as heart disease risk factors (38, 39), hypertension (39, 40), depression and anxiety (26, 41), diabetes (42), osteoporosis (43), and muscle weakness (44), as well as reduced immune function (45, 46), inflammation (47, 48), and autonomic imbalance (49). Many of these beneficial effects may happen via the influence of MM on the ANS (26, 50); see Figure 3.

### Study Aims

To evaluate COPD-related health factors in FA exposed to SHCS while flying, and to determine the effects of digitally delivered MM training on these factors.

There are three sub-aims, relating to the three main aspects of the study:

(1) To investigate the hypothesis that COPD-related pulmonary and other symptoms in SHCS-exposed FA are part of a syndrome of related disorders.
(2) To determine that MM practices are most suited to and effective for ameliorating pulmonary dysfunction and related health problems in FA exposed to SHCS.
(3) To conduct a randomized controlled trial (RCT) to determine whether this specifically adapted MM training can be effectively delivered digitally, providing the benefits of MM without face-to-face instruction being necessary.

## Methods

### Overall Structure of the Study

This study has three stages: Screening, Selection, and RCT. Screening will continue throughout the study and will gather data as well as serving to provide participants for the other Selection and RCT stages. The RCT will follow the Selection stage. All aspects of this study have been approved by the Dartmouth IRB. Additionally, this study is registered at ClinicalTrials.gov Identifier: NCT02612389. https://clinicaltrials.gov/ct2/show/NCT02612389/

#### Screening

To screen a cohort of FA exposed to SHCS for a range of biomarkers and symptoms to determine whether symptom clusters exist that suggest a syndromal entity.

##### Rationale

Flight attendants exposed to SHCS have been observed to display a wide range of respiratory, functional, inflammatory, autonomic, cardiovascular, affective, and other symptoms. The possible relationship between these symptoms has not previously been explored. We hypothesize that these symptoms may be correlated and form a syndromal entity.

#### Selection

To determine which of a variety of possible MM exercises are most effective in improving specific outcome measures, most enjoyable, and most likely to be practiced in a cohort of FA exposed to SHCS and having a degree of pulmonary dysfunction. This is done by conducting a 16-week in-person MM training program that is held in the Northeastern region of the US. Participants provide extensive feedback on the exercises.

##### Rationale

Meditative movement offers an inexpensive intervention that could benefit pulmonary symptoms and other related symptoms, through its effect on the ANS, cardiovascular, and immune systems. However, there is a large body of possible MM exercises, and there has been no investigation of the relative effectiveness, acceptability, and compliance among FA for the many MM exercises that are traditionally recommended for COPD-related symptoms. To our knowledge, no other study has attempted to evaluate a wide range of MM practices to determine which are most appropriate for the population being studied.

#### Randomized Controlled Trial

To develop a DVD or other digital form for delivering the selected exercises that have proven to most appropriate for this population, and to test by RCT the effectiveness of this digital format in improving specified outcome measures in a group of FA with pulmonary dysfunction and exposed to SHCS, as compared to a matched attention control group who will watch educational digital material of similar length.

##### Rationale

Selecting appropriate exercises and providing face-to-face instruction in MM offers significant difficulty due to the lack of trained teachers in most areas of the country and the practical demands of attending classes. A digitally delivered format would make this intervention much more accessible, and an RCT will evaluate the effectiveness of this intervention.

### Participants

#### Recruitment

Flight attendants were recruited from the Northeastern region and other areas of the US for the Selection stage; the Screening and RCT stages will involve recruitment throughout the continental US in locations convenient to data collection points. Primary outreach is through existing FA organizations and networks, social media, and FA publications, as well as in conjunction with the Harvard Flight Attendant Health Study. Social media support is also provided by Department of Health Behavior at the Roswell Park Cancer Institute in Buffalo, NY, USA.

#### Randomization and Blinding

The Screening and Selection stages of this study do not use a control group; blinding and randomization are, therefore, not necessary. In the RCT, participants will be stratified by gender, age, and severity of pulmonary dysfunction and randomized to an intervention groups and an attention control, using the covariate adaptive randomization method (51). This method accommodates recruitment and testing over an extended period of time. Full blinding of participants to intervention is not possible under these circumstances; however, all scoring of tests will be done by assistants blinded as to participant group.

#### Inclusion Criteria

The Screening stage of the study acts both to collect participant baseline data and to determine eligibility for the Selection and RCT stages.

All participants must be current or former non-smoker FAs employed by a US carrier for at least 5 years while smoking was permitted in the aircraft cabin. They must have a score of above 5 on the COPD assessment test (CATest); this will eliminate those with no detectable pulmonary issues. A score of 5 is a low score, indicating minimal level of pulmonary dysfunction.

Many FA exposed to SHCS have detectable pulmonary abnormalities despite not meeting Gold criteria for mild COPD (2); we wish to include this population, since they are likely at risk for the future development of COPD or its co-morbidities. We will eliminate any participants not meeting at least one of the following spirometric criteria: ratio of forced expiratory volume in 1 s to forced vital capacity (FEV1/FVC) < 0.70; OR forced expiratory flow (FEF) at 25–75%, volume <80% of predicted normal; OR FEF at 50%, volume <80% of predicted normal; OR FEF at 75%, volume <80% of predicted normal. We will follow procedures as described by Arjomandi et al. (2) for determining normal FEF values.

Participants must be lifetime non-smokers, defined as having smoked <100 cigarettes in their lifetime. They must have a device on which to listen to audio or watch video instruction and be willing to do so; for the RCT portion of the study, capacity to watch online or DVD videos is required. Those who are pregnant, or who plan to become pregnant during the study, are excluded. Patients with cognitive impairment, severe emotional problems, or who are unable physically to perform the exercises are excluded. Participants will be asked not to modify their lifestyle significantly during the study period apart from the practice required by the study. Participants must all sign informed consent forms.

#### Sample Size

The Screening stage of this study has no limit on number of participants. For the Selection and RCT stages of the study, we determined that, based on a predicted difference of 46 m in the 6-min walk test (6MWT) and an assessment of previous similar studies of MM for COPD, this study required a sample size of 20 participants in each group to achieve a statistical power of 80% at a significance level of 5%. To compensate for anticipated dropout, we recruited a total of 27 participants into the Selection group. On the basis of our experience of a high dropout and non-compliance rate in the Selection stage (~40%), we intend to recruit 120 participants for the RCT, 60 for each group.

### Interventions

The Screening stage of the study involves no intervention. In the Selection stage, the MM intervention is given in person in small groups (between 3 and 7), in 3-h classes, spaced 2–4 weeks apart, in locations convenient to the participants, for a total of 18 h of contact time over 4 months. Participant feedback will be solicited as to the subjective effectiveness, enjoyability, and likelihood of regular practice of each specific exercise.

The MM classes will be taught by one of the authors, Peter Payne, a Qigong teacher with over 40 years experience. In the classes, rather than teaching rote movements, the purpose and philosophy behind each exercise is explained. Suggestions are made as to how to set up a regular practice and how to integrate the exercises into everyday life. The participants are encouraged to ask questions and make comments on the exercises; all questions and comments are recorded in writing at the time. Individual attention is given to the needs of each participant, and written and audio explanations are also available to participants online. (Please see the Supplementary Material for the description of exercises from which we draw.)

The RCT stage of the study will involve the design of a digitally delivered intervention based on the exercises determined in the Selection stage to be most helpful. The intervention may be delivered on a DVD or through a web site. The goal will be to simulate as closely as possible the experience of attending a class through the development of innovative methods, such as computer animation, branching path selection, and user-controlled sequencing. Participants in the RCT will also have access to audio and written materials. Attention control subjects in the RCT will receive equivalent digitally delivered health education materials.

### Safety Considerations

Very few studies of MM have reported significant risks (52) and we do not anticipate any ill effects. Nevertheless, participants are instructed to discontinue the exercise immediately if they experience any of the following: dizziness, rapid or irregular heart-beat, sudden or excessive dyspnea, chest pain, significant pain anywhere in the body, and headache. In addition, during the classes we carefully monitor the participants for signs of distress, including facial paleness or redness, rapid breathing, sweating, or jerky movement. Any data gathered that suggest possible need for medical attention are drawn to the attention of the participants.

### Outcomes

These are the outcome measures to be used in the Screening stage of the study as well as to evaluate the effects of the MM practices in the Selection and RCT stages. The broad range of outcome measures will allow us to examine correlations between a wide range of variables representing a number of different physiological and psychological systems that may be involved in COPD and its co-morbid conditions.

#### Primary Outcome Measures

Six-minute walk test
Change in high sensitivity C-reactive protein (hs-CRP)

#### Secondary Outcome Measures

Blood pressure (BP) (pre- and post-6MWT),
Heart rate (HR) (pre- and post-6MWT),
Blood oxygen saturation,
Spirometry FVC, FEV1, FEF 25–75,
Compass 31 (Autonomic function self-report),
Zung Depression Inventory,
Zung Anxiety Inventory,
COPD Assessment Test (CATest),
FA Health Questionnaire,
Multidimensional Assessment of Interoceptive Awareness (MAIA),
Borg Dyspnea Scale,
Heart Rate Variability (HRV),
2 Ewing tests,
Urine analysis (melatonin and cortisol),
Saliva analysis (diurnal cortisol), and
Blood (fingerprick) analysis.

All tests are administered by trained researchers. Blood, urine, and saliva analysis provided by ZRT Laboratory, Beaverton, OR, USA.

### Outcome Measurement Details

#### General Health

##### Flight Attendant Health Questionnaire

This was developed by Dr. Eileen McNeely at the Harvard School of Public Health. Its use provides us with a health and FA-related occupational history and enables us to compare our study results with those of Dr. McNeely.

#### Functional Ability

Exercise endurance and tolerance is evaluated by the 6MWT followed by the Borg Dyspnea inventory. This is used as our principal outcome measure since it is a well-recognized measure of overall functional ability. The walk is conducted on an indoor level surface free of obstructions. Research assistants are on hand to assist participants with any difficulties. Maximum distance walked in 6 min is recorded (53–55). The difference in scoring from baseline to the end of study is calculated for each participant. *The Borg Dyspnea Inventory*, a well-established instrument for measuring respiratory distress, is administered following the 6MWT. The 6MWT is subject to variation due to motivation; the addition of the Borg instrument increases the reliability of the test.

#### Respiratory Health

##### The COPD Assessment Test

The CATest is used as an initial recruitment screening as well as an outcome measure. Volunteers must score above 5 for inclusion in the study. Its main purpose is to eliminate those with no appreciable respiratory dysfunction.

#### Spirometry

##### FEV1/FVC and Flow/Volume Curves

Forced expiratory volume in 1 s to forced vital capacity and Flow/Volume Curves are used as part of the inclusion criteria (see above under inclusion criteria), and to determine the degree of pulmonary dysfunction. We use specific validated spirometric cut points (56). We record FEV1/FVC ratio and Flow/Volume Curves using the EasyOne Plus Frontline spirometry system. FEV1/FVC is a standard measurement used to determine the GOLD grade of COPD. The Flow/Volume Curves indicate the degree of pulmonary dysfunction; participants with no detectable pulmonary dysfunction are excluded from the study. The differences in scoring from baseline to specific intervals are calculated for each participant.

#### Cardiovascular Measures

##### Blood Pressure and Heart Rate

Blood pressure and HR are measured at the beginning of each testing period and before and after the 6MWT by a trained technician using standard clinical instruments. *Cardiac R-R intervals* are recorded over the entire testing period (about 1½ h) using a commercially available system, the Holter myPatch 24-h single channel AMS3000 from DMS-Services, Los Angeles, CA, USA. This is a non-intrusive device, using two electrodes. Due to problems with availability, this measure was not included with some participants during the Selection stage of the study only. Blood oxygen saturation is also measured, using a Choicemmed Oxywatch Fingertip Pulse Oximeter.

#### Autonomic Condition

##### Heart rate Variability

Subjects are asked to sit quietly for 10 min. In post-analysis, the HR data collected during these periods is used to calculate HRV. HRV data will be analyzed using software provided by the manufacturer: CardioScan II, version 12.4.0054a, from DMS Software, Los Angeles, CA, USA. Time domain (SDNN, RMSSD) and frequency domain [power spectral density (PSD)] analysis methods will be used. Dr. Phyllis Stein, Director of the Washington University School of Medicine HRV Lab, will serve as a consultant on HRV analysis and interpretation. HRV measurements are only available for a subset of participants in the Selection stage due to delayed availability of HR monitors; monitors will be fully available during the RCT stage.

##### Ewing Tests

The Ewing tests evaluate cardiac autonomic function by measuring the response of HR or blood pressure to a physical challenge. We will administer two of the battery of five Ewing Tests: deep breathing heart rate challenge (DBHR) and lying to standing blood pressure challenge (LSBP). DBHR has been determined to be the most significant of the Ewing tests, carrying 80% of the significance. To measure this, participants will be asked to breathe deeply for three 1-min periods at a rate of six times a minute; post-analysis using recorded R-R intervals will enable evaluation of HR response to challenge, which is a measure of the integrity of the sympathetic branch of cardiac autonomic control. To measure LSBP, the systolic blood pressure change from supine to standing is measured. This gives an indication of cardiac parasympathetic function. Comparing the results from HR, BP, HRV, DBHR, and LSBP will allow a more accurate evaluation of the cardio-respiratory autonomic system than any one of them taken alone. Full Ewing test results are only available for a subset of participants due to problems with availability of the heart monitors.

##### COMPASS 31

In addition, we administer the COMPASS 31, a questionnaire for detecting dysfunction in many aspects of the ANS.

#### Affective State

##### Zung Anxiety and Depression Inventories

These evaluate the degree of affective disturbance. They are both well validated and widely used instruments. Since depression and anxiety are significant co-morbidities of COPD, and since we speculate that MM intervention will have an impact on them, the Zung inventories are relevant.

#### Interoceptive Awareness

##### The Multidimensional Assessment of Interoceptive Awareness (MAIA)

The Multidimensional Assessment of Interoceptive Awareness (MAIA) is a questionnaire that evaluates the degree of a person’s awareness of interoceptive cues, as well as his or her degree of comfort with these cues. We believe this is relevant to a person’s ability to benefit from the MM intervention.

#### Biomarkers

Biomarkers include urine, saliva, and blood analysis.

##### Blood Analysis

Capillary blood drops will be taken from the finger following fingerprick with a lancet, and deposited onto a blood spot card used specifically for dried blood spot (DBS) collections. Blood spots are dried on the filter card for at least 2 h before closing the cover and then further dried overnight at room temperature before storing the cards for shipment to ZRT Laboratory. Six-millimeter disks of blood spots are punched into 96-well plates and the blood extracted with buffers. Extracts are assayed for C-reactive protein (CRP), a marker of inflammation, as well as HbA1c (a marker of metabolic function), thyroid stimulating hormone, and Vitamin D.

##### Urine Steroid Hormone and Element Testing

Participants will be asked to provide first morning and then a bedtime urine samples: these are collected onto an absorbent paper strip and dried. They will be instructed in correct procedures for this. The sample will later be analyzed at ZRT Lab to determine by LC-MS/MS levels of urinary free cortisol, free cortisone, and melatonin, and by ICP-MS levels of iodine, bromine, selenium, arsenic, cadmium, and mercury (all normalized to creatinine).

##### Salivary Steroid Hormone Testing

Participants are instructed to provide four saliva samples over the course of a day. These samples will be stored frozen and batch-shipped to ZRT Laboratory and analyzed to determine diurnal cortisol levels as well as first morning estradiol, progesterone, testosterone, and dehydroepiandrosterone (DHEA) by LC-MS/MS.

Dried blood spots, dried urine (DU), and saliva samples are mailed to the researchers and stored at −70°C until they can be shipped in bulk to ZRT Labs for testing.

This extensive range of biomarkers will allow us to examine correlations between levels of function of a wide range of physiological systems and pulmonary dysfunction.

During the Selection and RCT stages of the study, feedback will be gathered from participants through written logs, and a questionnaire on Survey Monkey. Copies of the forms for the written logs and the Survey Monkey questionnaire are available as Supplementary Material.

### Statistical Methods

Descriptive statistics, including mean, SD, and frequency, will be used to describe and summarize baseline data. We will also compare distributions between intervention and control groups in the RCT stage using Wilcoxon rank-sum tests and chi-square tests at baseline. An intent-to-treat analysis will be carried out where missing data will be handled by multiple imputation approach, and linear regression models will be used to compare the intervention group with the control group in terms of the primary outcomes.

### Stepwise Methods

**Figure 4:**
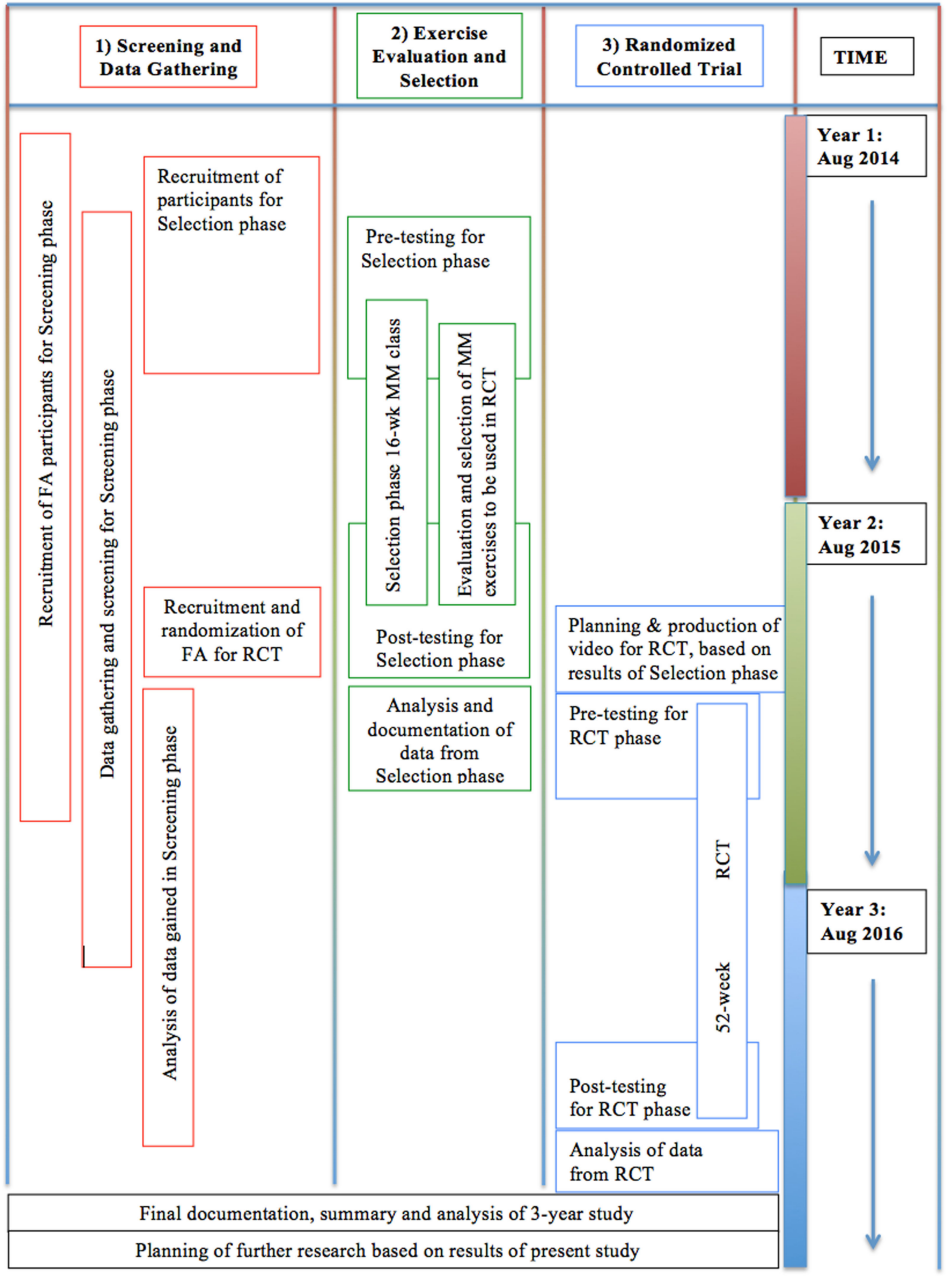
**Proposed timeline for the study**.

Stepwise methods are detailed in Figure 4.

### Anticipated Results

In the Selection and the RCT stages of this research, it is anticipated that subjects in the study will increase endurance as demonstrated by an increase in the 6MWT by margins that meet or exceed statistical significance. A general marker of inflammation, hs-CRP, is expected to decrease significantly. Consistent with evidence that MM may benefit the ANS, we expect to see a decrease in blood pressure. With the larger number of subjects in the RCT stage of the study, secondary measures are expected to provide data suggesting or discounting relationships between ANS function, mood, pulmonary, and immune system functions.

### Pitfalls, Artifacts, and Troubleshooting

A potential pitfall in this study is the difficulty in recruiting and retaining subjects. Our extensive network of contacts among FA will facilitate this process, as will our outreach through FA-connected social media. The recruitment process can be extended in time to assure adequate numbers. In addition, the researchers are prepared to travel to locations throughout the country as necessary.

Retention of subjects in the second and third stages is another concern. It is anticipated that retention will not exceed the 40% of the Selection stage of the study, as the exercises to be taught are simple and enjoyable. However we are allowing for up to 60% drop-out rate. Should dropout be more than anticipated, we will need to recruit more subjects, which should prove possible. We have maintained good communication with all subjects.

In the Selection stage of the study, any difficulties participants have had with the training, including safety-related concerns, were explored as a source of information concerning appropriate selection of exercises; this was one of the aims of this portion of the study.

The smoking status of study participants is queried during the recruitment and further during testing for each stage of this study. However, additional details of a smoking history have, in a few cases, emerged when other data had already been collected on these subjects. Outcomes from these subjects will be sequestered and reported separately from those who smoked less than 100 cigarettes in a lifetime.

Due to limited availability of equipment, the wearable ECGs were not used in the majority of pre-testing in the Selection stage. Changes in HRV are, therefore, not documented. However, other measures of cardiovascular autonomic function were made, including HR, BP, and changes in these measures in response to challenge. These will provide some information about possible autonomic changes.

In the Selection stage, we do not use a control group. Since the purpose of this portion is to determine that MM exercises are most effective and best tolerated, use of a control group would not be appropriate. This diminishes the power of results from the pre-and post-intervention tests in this portion as it will not be possible to determine with certainty whether results are due to attention, seasonal or other factors. However, the final RCT stage of the study, using an attention/health education control group, will provide a well-controlled validation of the effects of the selected MM exercises. It is, however, possible that positive results from the testing in the Selection stage, followed by negative results from the RCT stage, would reflect the ineffectiveness of digital delivery rather than the ineffectiveness of the intervention. In this case, an RCT testing in-person delivery would be indicated.

The 6MWT is known to be influenced by motivation. The use of the Borg Dyspnea Inventory is used as a control on this, as it indexes amount of effort, and every precaution has been taken by the researchers to standardize the administration of the test. Nonetheless, in the Selection stage, subjects may be more highly motivated in the post-test situation, and this could skew results. As mentioned above, the use of a control group in the RCT stage will provide a check on these results.

In the RCT stage, compliance is expected to be an issue. We plan to be in contact by email, phone, and digital questionnaire with all subjects on a weekly basis. We will follow up quickly any lapses in communication or reporting of difficulties, so as to minimize this issue. Such difficulties arising in this stage will also provide further information for future refinement of the delivery method and the selection of exercises.

## Discussion

We believe that the ANS may be a link between the various co-morbidities of COPD.

We speculate that COPD-related pulmonary and other co-morbid conditions appearing in FA exposed to SHCS may be part of a syndrome of interacting conditions related to disturbed autonomic function. The Screening stage of this study will provide data to test this speculation.

In addition to our primary outcome measures, we are gathering a wide range of data, including hormonal biomarkers, affective, and autonomic measures. This may allow for the formulation of hypotheses for future testing concerning the mechanisms of action of MM on pulmonary dysfunction and other health conditions. In preliminary data, we are seeing patterns of subclinical morbidities that may provide an improved picture of the health care needs of this unique group of workers. It, however, remains to be seen whether these patterns are simply age related or unique to this group and their exposure to SHCS.

Since MM has been shown to have beneficial effects on many of the co-morbid conditions of COPD, possibly by way of its regulating effect on autonomic functioning, this suggests that a single approach (MM) might target not only COPD-related respiratory symptoms but also many other COPD-related conditions. Future studies could determine whether MM could slow the progression of pre-COPD conditions to clinical COPD.

Previous trials of the effects of MM have relied on a preselected set of exercises, in some cases chosen for their traditional relevance to the condition being treated (57) but in most cases a traditional general set (30, 32–36). Our study is the first to involve a test period in which a wide range of MM exercises is evaluated for a specific population. We have already observed that participants have trouble setting aside time for regular practice. Our approach to MM includes a number of practices that can be integrated with the activities of daily life (rather than having to be practiced at a certain time), and we have found that this form of practice is more acceptable to this group of FA.

We experienced an unexpected level of dropout during the Selection stage of the study. Given that the average age of participants is 69, this should have been anticipated. We have found that the provision of exercises that can be integrated into daily life, such as simple standing, sitting, walking, and breathing practices, increases compliance and appears to reduce dropout. We believe that the emphasis on this kind of practice can make Asian practices more accessible and appealing to Westerners with their busy lifestyles.

Our study explores the possibility of administering an MM intervention by digital means. To our knowledge, no other study of MM has tested this possibility. Information gained from this aspect of the study may provide information about the most effective ways of conveying MM training digitally, as well as validating the effectiveness of MM for COPD-related pulmonary dysfunction. Should our principal hypothesis be supported, it would not only open the possibility of a safe, cost-effective intervention for those with pulmonary dysfunction, but also support the further exploration of digitally delivered MM for a wide range of conditions, especially those with a significant autonomic component.

### Trial Status

Recruitment for the Screening and evaluation portion of the study began in August 2014 and will continue for the duration of the study, until mid 2017. Comprehensive data have been gathered on more than 60 participants to date, and preliminary analysis has begun.

Recruitment for the MM exercise selection portion of the study began in October 2014 and will continue through October 2015. While 27 participants have been recruited so far into the Selection stage, there is concern for assuring that a statistically significant number will complete this stage of the study. No adverse events have been reported. Post-intervention testing began in July 20 2015, and is on going.

Preparations for the development of a digitally deliverable MM intervention are presently underway, involving developing video footage and planning. Recruitment for the RCT portion of the study began in October 2015.

## Author Contributions

PP: manuscript, research plan development, and MM instruction. DZ: data reporting and analysis, supervision of sample analysis, and manuscript. SF: manuscript, research plan, and supervision. MC: manuscript, administration, research plan development, and implementation.

## Conflict of Interest Statement

PP and MC in part receive compensation from teaching and consulting relating to Meditative Movement and MC from consulting on flight attendant health concerns; DZ is financially compensated by the lab that provides the biomarker analysis for the study. SF declares no conflict of interest.

## Appendix

**Table:**

ACRONYMS		
6MWT	Six-minute walk test	A standard test for assessing functional ability, the distance walked in 6 min
ANS	Autonomic nervous system	Portion of the nervous system controlling those physiological functions outside normal voluntary control
BP	Blood pressure	
COPD	Chronic obstructive pulmonary disease	Chronic disease involving obstruction of the small airways
CRP	C-reactive protein	Biomarker of inflammation
DBHR	Deep breathing heart rate	One of the battery of 5 Ewing tests for cardiac autonomic dysfunction; the most significant of the 5
DVD	Digital video disk	Standard vehicle for presenting video
EAC	Education activity control	A control group engaging in educational activity, in this case watching educational videos
FA	Flight attendants	
FAMRI	Flight attendant medical research institute	The foremost organization promoting research into medical problems affecting flight attendants
FEF	Forced expiratory flow	Spirometric measurement, flow of air during forced exhalation
FEV1	Forced expiratory volume one	A measure of lung function: the maximum volume of air breathed out in a forcible exhalation in 1 s
FVC	Forced vital capacity	A measure of lung function: the total volume of air breathed out in a complete forcible exhalation
GOLD	Global initiative on obstructive lung disease	A research and information group that is the principal global authority on the nature and treatment of COPD
HR	Heart rate	
HRV	Heart rate variability	Degree of variability over time (especially in synchrony with the breathing) of the R-R, or beat-to-beat, heart rate; since this variation of heart rate is under control of the parasympathetic nervous system, HRV offers a way of evaluating this aspect of ANS function
LSBP	Lying standing blood pressure	A Ewing test of the blood pressure response to going from lying to standing
MAIA	Multidimensional assessment of interoceptive awareness	A questionnaire designed to evaluate several dimensions of interoceptive awareness
MM	Meditative movement	A newly identified form of exercise characterized by a meditative state of mind, deep relaxation, movement, and attention to the breathing. Qigong and Yoga are the best known examples
NHANES	National health and nutrition examination survey	
PI	Principal investigator	
PSD	Power spectrum density	A measure of frequency distribution used to evaluate HRV
QOL	Quality of life	A general measure of the quality of a patients daily life
RCT	Randomized controlled trial	The premier form of research study in which participants are randomly assigned to control or intervention group for the purpose of objective assessment of an intervention
RMSSD	Root mean square standard deviation	A statistical method used in this study for the analysis of HRV data
SDNN	Standard deviation	A statistical method used in this study for the analysis of HRV data
SHCS	Second-hand cigarette smoke	Cigarette smoke inhaled by someone other than the smoker; ambient smoke not inhaled directly through the cigarette
