# Supplementary material for: Meditative Movement as a Treatment for Pulmonary Dysfunction in Flight Attendants Exposed to Second-Hand Cigarette Smoke: Study Protocol for a Randomized Trial
Source: Front Psychiatry. 2016 Mar 22;7:38. doi: 10.3389/fpsyt.2016.00038 (PMC4801846; doi:10.3389/fpsyt.2016.00038)
Supplement: Supplementary file 1 [file Data_Sheet_1.pdf]

Submitted as supplemental materials with manuscript:

Meditative Movement as a treatment for pulmonary dysfunction in flight attendants exposed to second-hand cigarette smoke: Study protocol for a randomized trial.

February 2016

Peter Payne, David Zava, Steven Fiering, Mardi Crane-Godreau
Contacts:
Mardi Crane-Godreau at Mardi.Crane@Dartmouth.edu
Peter Payne at Peter.Payne@Dartmouth.edu
Department of Microbiology and Immunology
Geisel School of Medicine at Dartmouth
HB 7936
1 Medical Center Dr.
Lebanon, NH 03756, USA

Office: 603-653-9970

Descriptions of the Qigong protocol and approach used in the *Meditative Movement as a treatment for pulmonary dysfunction in flight attendants exposed to second-hand cigarette smoke: Study protocol for a randomized trial* training includes philosophy, instructions on integrating Qigong into daily life, and instructions on specific practices. Below are descriptions of the Qigong training used and refined in the Selection Phase of this study. Video materials for the RCT phase will follow this basic approach and primarily focus on helping participants adapt the use of Qigong postures (lying, sitting, standing, moving and interacting) into activities of everyday living.

Body, Breath and Mind

Our body is always with us. Grounding, lightness and openness keep the body young and healthy; we can use MM to remind ourselves not to tighten or cramp the body, to move effortlessly, and to release bad habits that may contribute to premature ageing.

We are always breathing. The rhythm of our breath coordinates our movements and sustains our life. MM reminds us to let go of the breathing and to allow the breath to sink deeply into the belly as well as into the back. At any moment we can use the knowledge gained through MM breathing to replenish our energies and to release unwanted tension. A gentle awareness of the breath in the lower belly is one of the most important strategies for reducing stress and staying calm under challenge.

Our awareness is the most precious thing we have. MM shows us how to keep our awareness clear, open and fresh. We always have the choice to be more fully engaged with the present moment, and this is perhaps the most profound gift that MM offers.

Meditative Movement in Daily Life

Meditative Movement (MM) is not just an exercise to be practiced from time to time. Qigong shows you HOW to do everything we do in daily life, only better!

The real aim of MM is to integrate awareness and practice with daily life; daily practice is only a support for this goal, and not a goal in itself. If the demands of life do not allow you to take 10 minute or half an hour a day to practice, no problem! Every moment of your waking hours is an opportunity. Practicing MM in daily life simply means that you do whatever you are doing, but you use your knowledge of MM to become more centered and at ease in the midst of your life.

Standing, sitting, lying, moving, interacting —these are the five basic MM "postures". So any time in the day that you are doing any of these, you can practice MM.

Any time you are standing, like in line at the supermarket, at the copy machine, or waiting for coffee to brew— do the basic standing practice. Release your legs deep into the ground, your head and spine up to the sky, and let your shoulders broaden and your arms float lightly at your side. Feel the support of the ground, open your awareness to space, and feel your connection to everything around you.

When you are sitting, at the computer, at a meeting, or just waiting—feel your sit-bones and feet connecting with the earth; let your spine float up; your back can be supported by the chair or just floating up without support. Awareness can open up

all around you, and you can feel your breathing flowing full and free. Each moment of sitting is a moment of practice.

The Shakeout

The Shakeout is a quick and easy way of opening the joints, releasing the muscles, and opening your whole energy field. It can take 5 seconds or 20 minutes.
A) Start by shaking loose one hand; think of finger hand wrist joints and ask them all to open. Then do the elbow and the shoulder area in the same way. Now PAUSE, and take time to compare the feelings between the two arms. Then do the other arm. Feel the sensations wakened up in the arms! The arms radiate out into space from your heart.
B) Now the legs. Shake loose foot ankle toes; then the knee, loose and easy; finally the hip joint, shake from the pelvis/Center. PAUSE; feel the difference in the way the two legs connect you to the ground. Now do the other leg in the same way. PAUSE again, feel your connection to the ground.
C) Now imagine you have a long dinosaur tail, which hangs down deep into the earth. Shake the tail; lift it up behind, then drop it; tuck it under, then drop it. Feel the tail heavy and long. Feel how legs and tail work together to ground you. Next, feel your head poised lightly on top of your spine; this is your "observation turret". Gently wiggle your body so your head rocks gently around on top. Let it come to stillness and feel the poised alertness of your head as your awareness opens up 360 degrees in all directions.
Now shake and wriggle your whole spine and torso in every direction. If it feels safe to you, you can roll your body down forwards and let the spine and head hang and stretch out, the roll back up to straight; your head will be the last part of the body to return to upright. End with opening up the arms and leaning back to stretch the front, before settling back into the middle.
D) Feel your whole body, stable, light and open. Stop here, or continue to Basic Standing.

Basic Standing

Having loosened your whole body, you come to a natural effortless standing position. This is the most fundamental practice in Qigong. It is the most complete (though not the easiest!) releasing practice. You can practice almost anywhere. It is the basis for all movement practices. There are two ways of practicing: going through the checklist repeatedly, or just hanging out doing nothing. You will have learned the detailed instructions for bringing each part of the body into balance; this is a checklist to remind you.
Feet should be a comfortable distance apart, more or less parallel.
Come up on the "front triangle"; stretch the heels back and down till they just touch the ground. Don't lose contact for the ball of the big toe! Then press the heel onto the ground until you feel a firm contact. At this point the feet should feel a bit like suction cups; the weight is spread out evenly over the whole foot.
Knees should be soft, neither locked back nor bent. They should be able to wiggle lightly. (Sometimes we may need to have the knees bent slightly.)
Tail is dropped—not tucked, not arched.
Belly soft.
Back long and wide.
Chest and upper back broad and open.
Armpits open ("gorilla arms").
Hands soft and open
Back of the neck back, soft and long
Head poised as if lightly suspended from above. DO NOT lift the chin!
A subtle inner smile sends a gentle warmth all through and around you.
Feel that your legs and tail root you deeply into the earth; that your head floats lightly in space, being aware clearly in all directions; and feel your arms widening out from your heart, ready to touch the world around you.

Basic Sitting

Sitting is more of a challenge than standing, but it is something we do a lot and it is a good position for practicing the Basic Breathing.
First, you need a chair with a seat that is either flat or slopes slightly forward. The height of the chair should be such that your thighs slope slightly forwards when you are sitting in it.
Always begin with Standing, to get the feeling of a natural lengthening in your body. Position yourself in front of the chair and release the weight into the ground so that you gently lengthen and expand. Do not interfere with this openness during the sitting process. Find the crease of the groin, place your fingers there, and fold in at the hip joint as you let the knees bend. Let the eyes follow the motion of your body, do not try to keep them level. As the sit-bones land in the seat, let the body return to

upright, and feel that you are "standing" on your sit bones. From the pelvis up, there is no difference between sitting and standing.
Once you are sitting, sway your body gently back and forth to get a sense of the balance. Notice how as you sway back, at a certain point your abdomen tightens; then as you sway forwards, the abdomen lets go and the back tightens. Find the place between the two, where front and back are both released, and gently rest in this dynamic balance. It should be quite effortless, and not fixed at all. Since your body is aligned and balanced, you can let go completely without any tendency to collapse; in fact, as in standing, the more you let go the lighter you should feel From this stance you can begin the Basic Breathing practice.

Basic Breathing

Breathing is controlled instinctively by the deep centers of the brain, but we learn to interfere with it through bad habit patterns. The aim of Qigong breathing is to restore the natural pattern of instinctive breathing by letting go of interfering habits.
You can do this exercise sitting, standing or lying. Sitting is best. So start by sitting comfortably, spine long, resilient and straight. First, just notice the movement of your breath just as it is, with no effort to control or correct it. Where do you feel it? Chest, stomach, belly, back, sides? Is it fast or slow? Deep or shallow? Smooth or rough? What happens as you just observe it without interfering?
Now pay attention gently to the exhale. Do you allow the exhale to complete itself, or do you unconsciously stop it before the end? What happens if you allow the exhale to happen completely? (Don’t push it out!)
Perhaps you start to notice that the exhale prolongs itself a bit; and as it does so, the next inhale is a little fuller. Don’t force it! Don’t try to pull in a big breath! This just creates tension. Let go of the inhale; find that little natural pause at the end of the exhale. Notice that if you rest into that pause, the new inhale comes spontaneously, and fills you from below upwards. You start to feel the breath more in your belly, perhaps in your back and the sides of your ribs, and less in your upper chest and shoulders.
Continue to let go more and more, and gradually the instinctive brain centers take control. The breath, over time, becomes longer, deeper, smoother, subtler, and more powerful, like a vast tide moving slowly through you
The more the breath activates the belly, lower back and sides of the ribs, the more the neck, shoulders and upper chest can let go and soften, and the more the nervous system comes into balance.
he back is still straight, and well supported; this is a good alternative to balanced sitting.

How to do Qi movements (Drawing Down, Embrace the Earth, Pushing away, Gathering in)

The important thing about all the Qi movements is the *quality* of the movement, not the details of what moves where.
The first requirement is that the movement comes from the center. The shakeout helps with learning this. It really should feel as if the shifting, the opening and closing, coiling and uncoiling, of the center, is the source of the movement.
Secondly, the movement should be SOFT, SLOW, SMOOTH, and CIRCULAR. Softness is the most important; softer than soft, like cotton wool or a kitten’s fur.
Thirdly, feel the texture of the air you move through. Imagine the air is like water, or maple syrup! Notice how this makes you streamline your motion, and prompts you to use your whole body, not just empty arm motions. This also makes you follow the path of least resistance, the easiest, most natural path, similar to what would happen if your body were just swing free with natural momentum.
Finally, it should feel intentional. That is, there is a purpose to your motions, you are trying to do a certain thing; like calm yourself, move the energy down or up, make a boundary, and so on. The movements mean something, and they create something.
The breathing should just follow the movements naturally; don’t try to make the breathing coordinate with the motions, you will find this tends to happen naturally with time.
