# Supplementary material for: Meditative Movement as a Treatment for Pulmonary Dysfunction in Flight Attendants Exposed to Second-Hand Cigarette Smoke: Study Protocol for a Randomized Trial
Source: Front Psychiatry. 2016 Mar 22;7:38. doi: 10.3389/fpsyt.2016.00038 (PMC4801846; doi:10.3389/fpsyt.2016.00038)
Supplement: Supplementary file 2 [file Data_Sheet_2.pdf]

Submitted as supplemental materials with manuscript: **Meditative Movement as a treatment for pulmonary dysfunction in flight attendants exposed to second-hand cigarette smoke: Study protocol for a randomized trial.**

February 2016

Peter Payne, David Zava, Steven Fiering, Mardi Crane-Godreau
Contacts:
Mardi Crane-Godreau at Mardi.Crane@Dartmouth.edu
Peter Payne at Peter.Payne@Dartmouth.edu
Department of Microbiology and Immunology
Geisel School of Medicine at Dartmouth
HB 7936
1 Medical Center Dr.
Lebanon, NH 03756, USA
Office: 603-653-9970

Supplemental Materials: Survey Monkey Questionnaire

We have set up this web based survey to make it easier to provide feedback in the Meditative Movement study. This is a much shorter version of the paper questionnaire.

Feel free to **answer ONLY those questions that seem relevant to your situation.** Some questions relate to new materials that you might not have practiced yet.

Only 2 questions require answers, your ID and todays' date.

***1. Please enter your study ID number. (If you do not recall your ID number enter your initials.)**

***2. Please enter today's date.**
Date / Time MM/DD/YYYY

3. How much time did you spend 'doing' any of the exercises today? Please include time spent when you combined your exercises into daily activities.

4. How much total time did you spend on any of these exercises today? Include any time that you were able to do these as part of activities of normal living.
Basic breathing
Basic standing
Basic sitting
Hissing breath
Shake out
Drawing Down
Embracing the Earth
Patting and Stroking
Pushing Away Meditation
Field of Awareness Meditation
Lying With and Without Form

5. What meditative movement techniques do you most enjoy doing?

6. Which of the meditative movement techniques are you most likely to do?

7. Which of the techniques seem to be most helpful for you? How are they helpful?

8. How many hours of sleep did you get on average, in each 24 hour period over the last week?
Hours of sleep?

9. If you have missed doing the survey for more than one day, can you give us a brief summary of how frequently and for how long you did the exercises since you last reported your activity.

10. Have you accessed the audio files for class at www.FAHealth.org/audio/

11. Please use this space to record any unusual experiences, sensations positive or negative. IF YOU HAVE ANY CONCERNS RELATED TO THE STUDY, CALL OUR OFFICE AND STOP DOING THE EXERCISES UNTIL THE CONCERN IS RESOLVED. OFFICE NUMBER IS 603-653-9972.

12. Have you had any health concerns this week? Especially please tell us about any respiratory illness.

13. Do you have any other comments, questions, or concerns?

14. In your opinion, could the audio files deliver adequate training for teaching MM methods to flight attendants? What is needed to make these useful and adequate for study subjects?
Are audio files useful and adequate?

THANK YOU FOR YOUR PARTICIPATION.
